# Cancer of the larynx in non-smoking alcohol drinkers and in non-drinking tobacco smokers

**DOI:** 10.1038/sj.bjc.6600469

**Published:** 2002-08-27

**Authors:** C Bosetti, S Gallus, S Franceschi, F Levi, M Bertuzzi, E Negri, R Talamini, C La Vecchia

**Affiliations:** Istituto di Ricerche Farmacologiche ‘Mario Negri’, Milan, Italy; Field and Intervention Studies Unit, International Agency for Research on Cancer, Lyon, France; Registre Vaudois des Tumeurs, Institut Universitaire de Médicine Sociale et Préventive, Lausanne, Switzerland; Servizio di Epidemiologia, Centro di Riferimento Oncologico, Aviano, Italy; Istituto di Statistica Medica e Biometria, Università degli Studi di Milano, Milan, Italy

**Keywords:** laryngeal cancer, smoking, alcohol drinking, case–control study

## Abstract

The separate effect of alcohol and tobacco on laryngeal cancer was analysed in two case–control studies from Italy and Switzerland, comprising 40 non-smoking and 68 non-drinking cases, and 160 non-smoking and 161 non-drinking controls. The multivariate odds ratio was 2.46 for heavy drinkers non-smokers, and 9.38 for current smokers non-drinkers.

*British Journal of Cancer* (2002) **87**, 516–518. doi:10.1038/sj.bjc.6600469 www.bjcancer.com

© 2002 Cancer Research UK

## 

Alcohol drinking and tobacco smoking increase the risk of laryngeal cancer, and show a multiplicative effect on risk (Iarc, 1986, 1988; Tuyns *et al*, 1988; Franceschi *et al*, 1990; Sankaranarayanan *et al*, 1990; Dosemeci *et al*, 1997). In developed countries, laryngeal cancer is extremely rare in individuals who do not smoke and do not drink alcohol and information on risk in such people is therefore limited.

A hospital-based US study found an association with tobacco in non/moderate alcohol drinkers, but had no information on alcohol in non-smokers (Wynder *et al*, 1976). A case–control study from Canada, reported an increasing risk of laryngeal cancer in relation to alcohol drinking in non-smokers, based, however, on three case–control pairs only (Burch *et al*, 1981). A case–control study conducted in Italy, Spain, Switzerland and France reported odds ratios (OR) of 1.7 for ⩾80 g day^−1^ of alcohol among nine non-smoking cancer cases of the hypopharynx and epilarynx, and of 6.7 for ⩾40 g day^−1^ of alcohol among 22 non-smoking cancer cases of the endolarynx (Tuyns *et al*, 1988). A Polish study found a strong association with number of cigarettes among non-vodka drinkers, but the reference category was based on a single case (Zatonski *et al*, 1990).

## PATIENTS AND METHODS

The separate effect of alcohol and tobacco on laryngeal cancer risk was analysed in the combined data of two case–control studies, including a uniquely large number of non-smoking and non-drinking subjects. The first study, conducted between 1986 and 1992 in the province of Pordenone and the greater Milan area, northern Italy, included 162 subjects with incident, histologically confirmed cancer of the larynx (Franceschi *et al*, 1990; La Vecchia *et al*, 1990). The second one was conducted between 1992 and 2000 in the province of Pordenone and the greater Milan area, Italy, and in the Swiss Canton of Vaud, on 527 incident, histologically confirmed laryngeal cancer cases. Forty non-smoking cases (median age 60 years, range 30–72) and 68 non-drinking ones (median age 61 years, range 42–74) were identified in the two studies, and included in the present analysis. Non-smoking cases included 20 glottis, two supraglottis and 18 other or unspecified laryngeal cancers; non-drinking ones included 21 glottis, 12 supraglottis and 35 other or unspecified laryngeal cancers. They were matched on study, sex, age and study center to 160 non-smoking controls (median age 60 years, range 31–79) and 161 non-drinking controls (median age 59 years, range 40–77), selected from a database of 4781 patients, admitted to the same network of hospitals as cases, for a wide spectrum of acute, non-neoplastic conditions, unrelated to alcohol consumption and tobacco use. Among non-smoking controls, 25% had traumatic conditions, 24% non-traumatic orthopaedic disorders, 29% acute surgical conditions, and 22% miscellaneous other illnesses. Among non-drinking controls, 14% had traumatic conditions, 31% non-traumatic orthopaedic disorders, 34% acute surgical conditions, and 21% miscellaneous other illnesses.

Structured questionnaires were administered to study subjects during their hospital stay; information was collected on socio-demographic characteristics, anthropometric variables, and various lifestyle habits, including tobacco smoking and alcohol drinking. The questions on alcohol included the number of days per week each alcoholic beverage (i.e. wine, beer, hard liquors and spirits) was consumed, the daily number of drinks, and the duration of the habit. One drink corresponded to 125 ml of wine, 330 ml of beer, and 30 ml of hard liquors and spirits (i.e. about 12 g of alcohol). Non-drinkers were individuals who had abstained from drinking any type of alcoholic beverages lifelong. Information on smoking included smoking status (never, ex and current smoker), number of cigarettes and/or cigars habitually smoked per day, grams of tobacco for pipe smoking, age at starting and duration of the habit. Non-smokers were subjects who had never smoked at least one cigarette per day for at least 1 year. Ex-smokers were individuals who had abstained from any type of smoking for at least 12 months at the time of cancer diagnosis or interviews (for controls). Information on tobacco and alcohol was satisfactorily reproducible (D'Avanzo *et al*, 1996) and valid (Ferraroni *et al*, 1996).

Odds ratios (OR) and 95% confidence intervals (CI) were estimated using conditional multiple logistic regression models (Breslow and Day, 1980), adjusted for education, in addition to the matching variables. Tests for trend were based on the likelihood–ratio test between the models with and without a linear term for each variable of interest.

## RESULTS

Table 1Table 1

**Table 1:**
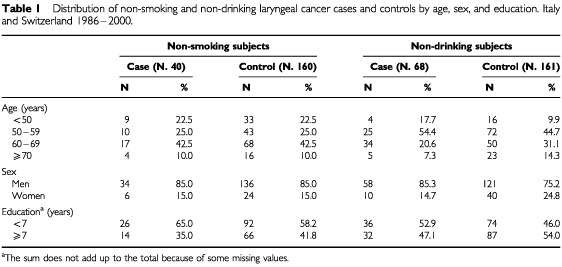
Distribution of non-smoking and non-drinking laryngeal cancer cases and controls by age, sex, and education. Italy and Switzerland 1986–2000

shows the distribution of non-smoking and non-drinking laryngeal cancer cases and corresponding controls according to age, sex, and education.

Table 2Table 2

**Table 2:**
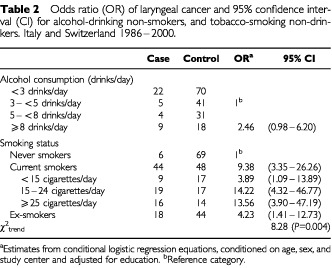
Odds ratio (OR) of laryngeal cancer and 95% confidence interval (CI) for alcohol-drinking non-smokers, and tobacco-smoking non-drinkers. Italy and Switzerland 1986–2000

gives the OR of laryngeal cancer for alcohol in non-smokers and for tobacco in non-drinkers. In non-smokers, the OR was 2.46 (95% CI: 0.98–6.20) for ⩾8 drinks of alcohol per day, compared to less than eight. There was no evidence of an increased risk for lower levels of alcohol intake. When the reference category was set at subjects drinking <3 drinks per day, the OR were below unit until seven drinks, and the trend in risk was not significant. In non-drinkers, the ORs were 9.38 (95% CI: 3.35–26.26) for current smokers and 4.23 (95% CI: 1.41–12.73) for ex-smokers, as compared to never smokers. The OR increased significantly (*P* for trend=0.004) with number of cigarettes smoked and was 13.56 (95% CI: 3.90–47.19) for ⩾25 cigarettes per day.

## DISCUSSION

This uniquely large dataset confirms a strong role of tobacco on laryngeal cancer risk, even in non-drinkers (Wynder *et al*, 1976; Burch *et al*, 1981). It also shows that elevated alcohol consumption appears to increase the risk in the absence of smoking (Burch *et al*, 1981; Tuyns *et al*, 1988). In contrast with cancers of the oral cavity (Talamini *et al*, 1998) and oesophagus (La Vecchia *et al*, 1999), no excess risk was observed for moderate alcohol intake.

The strong direct association in non-drinking current smokers further supports the prominent carcinogenic effect of tobacco *per se* on laryngeal cancer risk (Tavani *et al*, 1994). Tobacco is known to contain various carcinogenic substances, which come in contact with the laryngeal epithelium and act during the various phases of the process of carcinogenesis (Iarc, 1986).

An independent role of alcohol from that of tobacco is less striking, although plausible. Alcohol has not been shown to have a direct carcinogenic action on the larynx, as well as other upper digestive and respiratory sites, and has been generally thought to act only as a cancer promoter (Doll *et al*, 1999). Furthermore, even in smokers alcohol is a less strong risk factor than tobacco, and only the upper part of the larynx has direct contact with alcohol. However, alcohol contains metabolites with a carcinogenic potential, and it may favour penetration of other carcinogens, including those in foods, through local irritation and epithelial lesions (Iarc, 1988).
